# Identification of a novel *TSC1* gene variant in a patient with atypical vitiligo‐like skin lesions: Unveiling the hidden tuberous sclerosis complex

**DOI:** 10.1002/mgg3.2403

**Published:** 2024-03-04

**Authors:** Linli Liu, Yanbo Wang, Zhengzhong Zhang, Chunshui Yu, Jin Chen

**Affiliations:** ^1^ Department of Dermatology The First Affiliated Hospital of Chongqing Medical University Chongqing China; ^2^ Department of Dermatology Suining Central Hospital Suining Sichuan China; ^3^ Department of Dermatology Langzhong People's Hospital Nanchong Sichuan China; ^4^ Department of Dermatology Affiliated Hospital of North Sichuan Medical College Nanchong Sichuan China

**Keywords:** minigene assay, splicing variant, *TSC1*, tuberous sclerosis complex

## Abstract

**Background:**

Tuberous sclerosis complex (TSC), an autosomal‐dominant disorder, is characterized by hamartomas affecting multiple organ systems. The underlying etiology of TSC is the pathogenic variations of the *TSC1* or *TSC2* genes. The phenotype variability of TSC could lead to missed diagnosis; therefore, the latest molecular diagnostic criteria for identifying a heterozygous pathogenic variant in either the *TSC1* or *TSC2* gene filled this gap. Furthermore, the pathogenicity of numerous variants remains unverified, potentially leading to misinterpretations of their functional consequences.

**Methods:**

In this study, a single patient presenting with atypical vitiligo‐like skin lesions suspected to have TSC was enrolled. Targeted next‐generation sequencing and Sanger sequencing were employed to identify a pathogenic variant. Additionally, a minigene splicing assay was conducted to assess the impact of *TSC1* c.1030‐2A>T, located in intron 10, on RNA splicing.

**Results:**

A novel *TSC1*: c.1030‐2A>T heterozygosis variant was identified in intron 10. In vitro minigene assay revealed that the c.1030‐2A>T variant caused exon 11 skipping, resulting in a frameshift in the absence of 112 base pairs of mature messenger RNA and premature termination after 174 base pairs (p.Ala344Glnfs*59).

**Conclusion:**

The detection of this novel pathogenic *TSC1* variant in the patient with atypical vitiligo‐like skin lesions enrolled in our study ultimately resulted in the diagnosis of TSC. As a result, our study contributes to expanding the mutational spectrum of the *TSC1* gene and refining the genotype–phenotype map of TSC.

## Graphical abstract

We revealed the impact of a novel *TSC1* splicing variant on RNA splicing via a minigene assay, thus broadening the mutational spectrum of the *TSC1* gene.

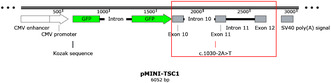

## 1 INTRODUCTION

Tuberous sclerosis complex (TSC, OMIM 191100) is an autosomal‐dominant disorder marked by the growth of noncancerous tumors in multiple organs, such as the brain, kidneys, heart, skin, and lungs (Henske et al., 2016). However, more than two‐thirds of TSC patients are sporadic cases (Hung et al., 2006). Mutations in the *TSC1* (OMIM, #605284) and *TSC2* (OMIM, #613254) genes, which encode hamartin and tuberin, respectively, and regulate cell growth and proliferation, are responsible for this disease (Randle, 2017).The phenotype of TSC is highly variable, even within the same family. Due to this phenotypic variability, diagnosing TSC can be challenging, especially in young individuals or those with subtle manifestations (Roach & Sparagana, 2016). This phenotypic variation could be attributed to the randomness of second‐hit events in TSC or as‐yet‐unidentified genetic modifiers (Lyczkowski et al., 2007).

Genetic testing has become an independent diagnostic criterion, with a heterozygous pathogenic variant in the *TSC1* or *TSC2* gene detected through molecular genetic testing (Northrup et al., 2021).

While the Sanger sequencing analysis has a historical role in genetic analysis, it is limited in its ability to screen large regions of the human genome in a single reaction (Crossley et al., 2020). Mutations in the *TSC1* and *TSC2* genes encompass a range of types, including missense, splice site mutations, small deletions, and nonsense mutations, without a discernible hotspot mutation site (Touraine et al., 2022). Next‐generation sequencing (NGS) is widely recognized as the most potent molecular analysis technique, aiming for high coverage of enriched regions that include all exons and a substantial portion of introns in the *TSC1* and *TSC2* genes (Northrup et al., 2021). These techniques have revolutionized areas such as precision medicine, hereditary disorders, and clinical diagnosis (Ali Khan, 2021). However, despite the power molecular strategies in identifying mutations in *TSC1* and *TSC2*, a significant proportion of clinically diagnosed TSC patients remain without identified mutations (Northrup et al., 2021). This highlights the need for alternative approaches such as the minigene assay, which can efficiently assess the impact of sequence variants on splicing, aiding in the identification of sequence variants with unknown biological and clinical significance (Gaildrat et al., 2010).

This study presents a sporadic patient manifested with atypical vitiligo‐like skin lesions, clinically suspected as TSC. Targeted NGS and Sanger sequencing unveiled a hitherto unreported heterozygous variant, *TSC1*: c.1030‐2A>T, located within intron 10 of the *TSC1* gene. The subsequent minigene assay confirmed that this aberrant splicing variant resulted in exon 11 skipping during messenger RNA (mRNA) processing (p.Ala344Glnfs*59), leading to the production of an abnormal coding protein and providing insights into the underlying pathogenic mechanism in this TSC patient. This study underscores the importance of genetic testing and alternative molecular analysis techniques in diagnosing TSC, especially in cases with atypical clinical presentations.

## 2 PATIENT AND METHODS

### 2.1 Ethical compliance

We obtained written informed consent from the patient, and the Ethical Committee of The First Affiliated Hospital of Chongqing Medical University granted ethical approval for this study.

### 2.2 Case description

A 51‐year‐old Chinese male presented with atypical vitiligo‐like skin lesions for nearly 50 years without any itching or pain. He was born with normal birth weight, following a full‐term, uneventful pregnancy to a non‐consanguineous, healthy couple. He had a suspected history of epilepsy. The patient exhibited typical developmental milestones. No family history of similar clinical manifestations was reported.

**FIGURE 1:**
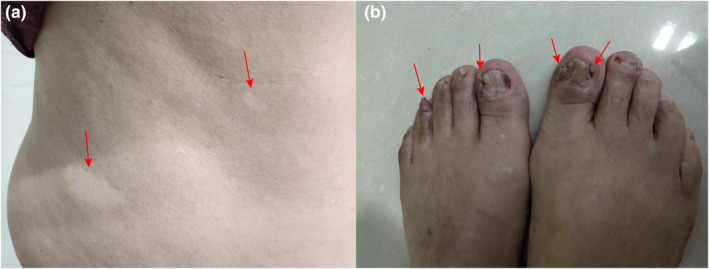
Clinical manifestations of the patient with suspected TSC. (a) Two hypomelanotic macules. (b) Ungual fibromas.

Upon clinical examination, the patient displayed typical growth parameters, weighing 71 kg and measuring 168 cm in height. The dermatological assessment revealed two hypomelanotic macules on the torso skin. Moreover, multiple papules were observed surrounding the nails, which caused nail deformation and damage (Figure 1). Cardiac and pulmonary examinations revealed no abnormal findings. The patient exhibited normal intelligence and vision. Biochemical analysis and abdominal color ultrasound showed no abnormal findings.

### 2.3 Targeted next‐generation sequencing and data analysis

Genomic DNA was extracted from blood samples using the Qiagen DNA Blood Midi/Mini kit (Qiagen GmbH, Hilden, Germany). After rigorous quality control of the DNA samples, sequencing was performed on the Illumina HiSeq X Ten platform (Illumina, San Diego, CA, USA) with an average sequencing depth exceeding 200× and a Q30 > 90%. Briefly, the patient underwent testing utilizing a panel encompassing 541 genes associated with monogenic hereditary diseases. Sequence reads were aligned to the GRCh37/hg19 reference genome using BWA software. Less reliable variant calls and common variants were filtered out through rigorous bioinformatics analysis. Variant pathogenicity was assessed following the standards and guidelines established by the American College of Medical Genetics and Genomics (ACMG).

### 2.4 Sanger sequencing

The potential *TSC1* variant identified through NGS (NM_000368.4: c.1030‐2A>T) was validated in the patient using polymerase chain reaction (PCR). Primers were meticulously designed based on the *TSC1* (GenBank NM_000368.4) sequence using PRIMER 5 software for PCR amplification. The following variant‐specific primers were employed: Forward 5′‐TAGTGTGCCTGCTCTCTCCT‐3′ and Reverse 5′‐CGGCAGATCACACCTTGAGA‐3′. Following purification, the PCR products were subjected to sequencing using the ABI PRISM 3730 Genetic Analyzer.

### 2.5 In vitro splicing assay

A minigene splicing assay was used to evaluate the impact of the *TSC1* intron 10 c.1030‐2A>T variant on RNA splicing. A 6052 bp fragment of the *TSC1* gene, encompassing exons 10–12 and introns 10–11, was utilized to construct minigenes. The wild‐type and mutant sequences were synthesized using NheI/NotI restriction sites. The target gene fragment was cloned into a plasmid vector. Design primers that flanked the target gene fragment, including its introns and exons, were as follows: Forward 5′‐catggacgagctgtacaagCTCGAGGggtgtgctacttctaccc‐3′, Reverse 5′‐CGCGGTACCGTCGACTGCAGAATTCCTActtcctggggggtgtgact‐3′, mutant Forward 5′‐acttcccTggctactctttggagcccat‐3′, and mutant Reverse 5′‐caaagagtagccAgggaagttaataaagtacatcagcagtggc‐3′. Positive clones were sent to the Shanghai Biotechnology Company for sequencing to select the correct minigene plasmid.

The 293 T cell lines were cultured in Dulbecco modified Eagle medium supplemented with 10% fetal bovine serum (10099141C, Gibco) and maintained at 37°C with 5% carbon dioxide in a humidified incubator. Cell transfection was executed using Lipofectamine 2000 Transfection Reagent (11668–019, Invitrogen), according to the manufacturer's protocol.

Forty‐eight hours post‐transfection, 293 T cells were harvested, and total RNA was extracted utilizing TRIzol reagent (9109, TAKARA). Reverse transcription‐PCR was performed using a pair of primers, MiniRT‐F (5′‐CCCGACAACCACTACCTGAG‐3′) and MiniRT‐R (5′‐ACCTCTACAAATGTGGTATGGC‐3′). The resulting PCR products were visualized via electrophoresis on 2% agarose gel and confirmed by Sanger sequencing.

## 3 RESULTS

**FIGURE 2:**
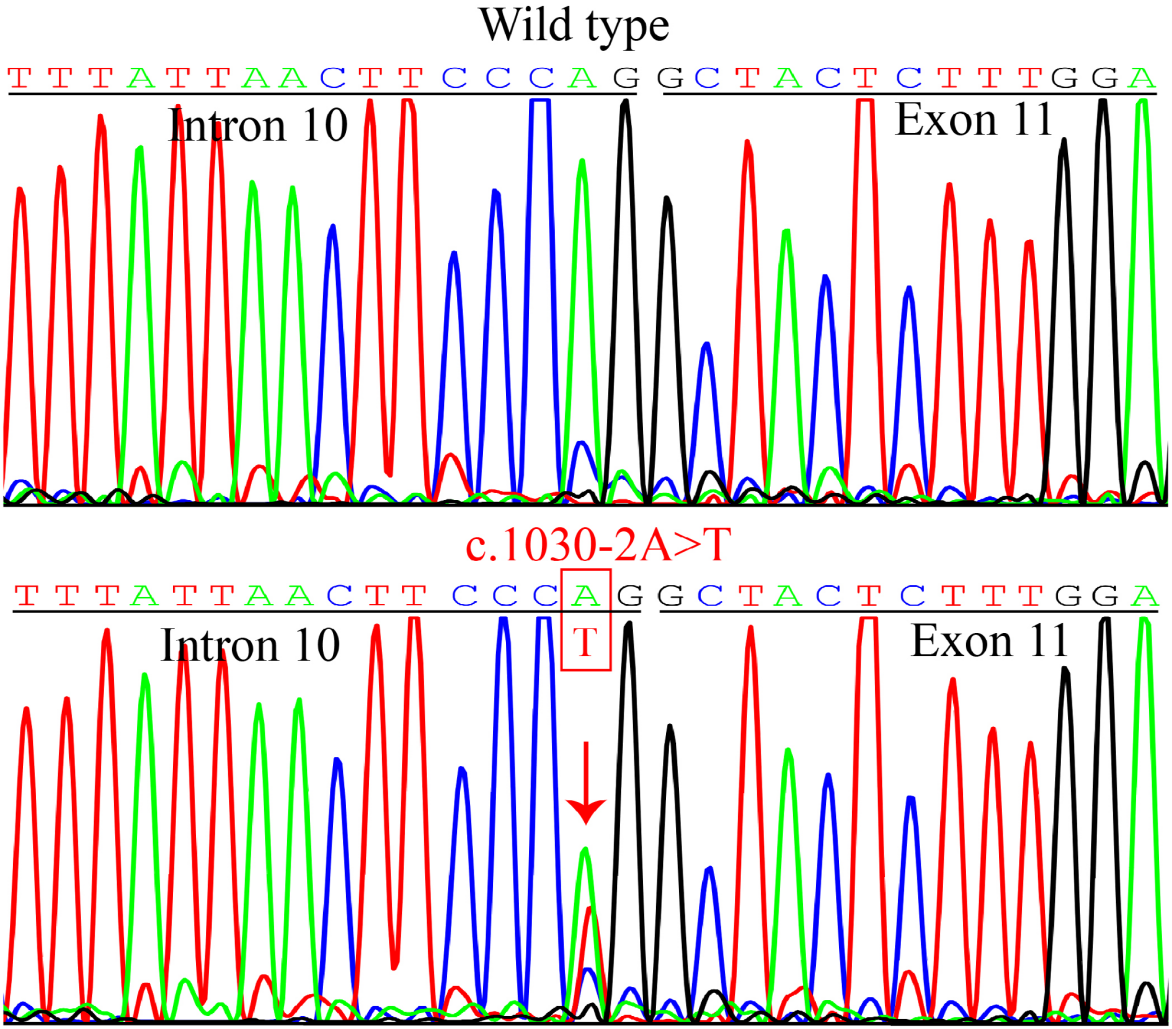
Heterozygous *TSC1*: NM_000368.4: c.1030‐2A>T variant (arrow) was identified in the patient with suspected TSC.

Targeted NGS in the patient revealed a heterozygous candidate variant in *TSC1*: c.1030‐2A>T in intron 10. Sanger sequencing verified this novel heterozygous *TSC1* splice variant, which was not found in the patient's family members or the 100 unrelated controls (Figure 2). This was considered novel because it was not present in the ExAC, 1000G, or HGMD databases.

**FIGURE 3:**
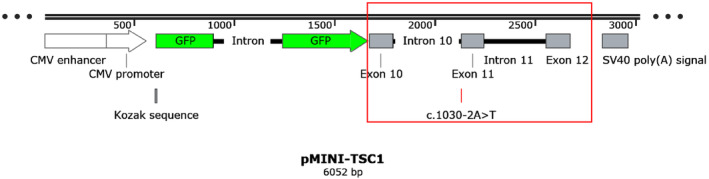
Structure diagram of pMINI‐*TSC1* The red box shows exon 10 to 12 fragments of *TCS1* gene, containing the designation c.1030‐2A>T variant.

**FIGURE 4:**
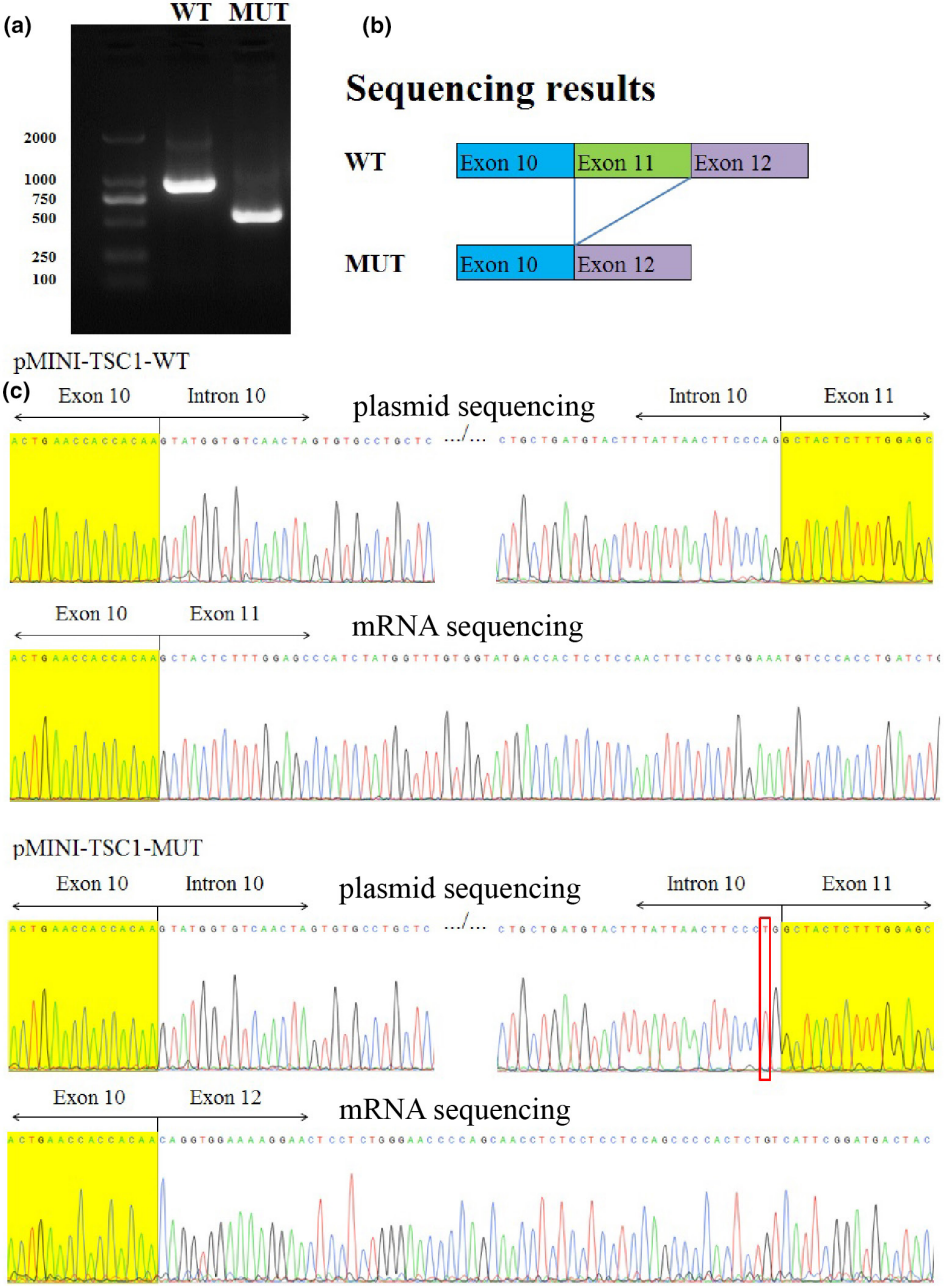
Transcript analyses of pMINI‐*TSC1*. (a)Electrophoresis showed that the variant c.1030‐2A>T led to a subtly smaller TSC1 mRNA transcript than the wild‐type clone. (b) Sequencing result of c.1030‐2A>T transcripts. (c) Plasmid sequencing and mRNA sequencing of pMINI‐*TCS1* showed that the pMINI‐*TCS1*‐WT was considered as expected normal splicing, while the mRNA sequence of pMINI‐*TCS1*‐MUT was changed. Abnormal splicing lead to exon 11 skipping during mRNA cutting, resulting in frameshift in the absence of 112 bp of mature mRNA, and premature termination after 174 bp, resulting in abnormal coding protein.

To assess the effect of *TSC1*: c.1030‐2A>T on RNA splicing, a minigene splicing assay was performed. The structure of pMINI‐TSC1 was constructed, containing the designation c.1030‐2A>T variant (Figure 3). Electrophoresis showed that the variant c.1030‐2A>T led to a subtly smaller *TSC1* mRNA transcript than the wild‐type clone. Plasmid sequencing and mRNA sequencing of *pMINI‐TCS1* showed that the *pMINI‐TCS1‐WT* was considered as expected normal splicing, while the mRNA sequence of *pMINI‐TCS1‐MUT* was changed. Abnormal splicing led to exon 11 skipping during mRNA cutting, resulting in a frameshift in the absence of 112 bp of mature mRNA and premature termination after 174 bp (p.Ala344Glnfs*59), resulting in abnormal coding protein. This reveals that this variant may be a crucial cog in the pathogenesis (Figure 4).

The correlation between the clinical data of the patient, including atypical vitiligo‐like skin lesions, ungual fibromas, and a suspected history of epilepsy, with the identified *TSC1* gene variant strongly supports the pathogenicity of the variant in causing the observed phenotype. In line with the ACMG guidelines (Richards et al., 2015), this variant was unequivocally classified as pathogenic (PVS1 + PM2 + PM6), solidifying its role in the pathogenesis of the patient's condition.

## 4 DISCUSSION

TSC is a genetic multisystem disease with variable manifestations that predominantly involve the skin. The main dermatological manifestations in TSC patients include hypomelanotic macules (ash‐leaf spots), facial angiofibroma, shagreen patches, and ungual fibromas (Ebrahimi‐Fakhari et al., 2017). The 2012 TSC criteria emphasize the importance of dermatological findings, accounting for 4 out of 11 major criteria and 3 out of 6 minor criteria (Teng et al., 2014). Given that dermatological manifestations may occur in every TSC‐affected individual, comprehensive dermatologic evaluation is crucial when assessing these lesions. Moreover, owing to insufficient clinical evidence and mild manifestations, approximately 20% of TSC proband have been missed (Togi et al., 2022). As an independent genetic diagnostic standard, genetic testing plays a crucial role in the diagnosis of TSC (Teng et al., 2014).

The main manifestation in this patient was atypical vitiligo‐like skin lesions, which were present for 50 years. He had a suspected history of epilepsy in his early years but showed no recurrence since then. Further detailed dermatological examination revealed important findings of papules around the nails, which were clinically diagnosed as ungual fibromas. Two hypomelanotic macules found in the patient did not fulfill the major criteria of TSC, which specifically requires more than three hypomelanotic macules (Teng et al., 2014). Thus, the patient was suspected to have TSC, and genetic detection was performed. A novel heterozygous variant, *TSC1*: c.1030‐2A>T, located in intron 10 of the *TSC1* gene was identified in this patient. This variant was not present in the patient's family members or 100 unrelated controls, and further analysis revealed its absence in well‐established genetic variant databases, such as ExAC, 1000G, or HGMD. Without sufficient phenotype, accurate prediction of variant pathogenicity is crucial. Hence, we employed a minigene splicing assay to assess the functional impact of the *TSC1*: c.1030‐2A>T variant on RNA splicing. This assay demonstrated that the variant exhibited abnormal splicing. This aberrant splicing event led to exon 11 skipping during mRNA cutting, resulting in a frameshift in the absence of 112 bp of mature mRNA and premature termination after 174 bp (p.Ala344Glnfs*59). The skipping of whole exon 11 is predicted to lead to defective tuberin production and severe impairment of the normal hamartin–tuberin interaction, thus resulting in an upregulation of the mammalian target of rapamycin pathway and manifestation of TSC. These findings strongly suggest that the *TSC1*: c.1030‐2A>T variant was a critical mechanism contributing to the pathogenesis in this patient. According to the ACMG guidelines, the *TSC1*: c.1030‐2A>T variant is pathogenic (PVS1 + PM2 + PM6) (Richards et al., 2015). This patient was eventually diagnosed with TSC despite the insufficient clinical manifestations. Genetic studies have identified a wide range of pathogenic variations within the *TSC1* and *TSC2* genes that contribute to TSC. Conventional molecular testing for TSC, such as Sanger sequencing, involved detecting point mutations in the coding regions and intron/exon boundaries of *TSC1* and *TSC2* genes. However, approximately 10%–15% of patients diagnosed with TSC do not have a detectable mutation on traditional genetic testing (Northrup et al., 2021). Some individuals with clinical signs of TSC have low‐level mosaic pathogenic variants, intronic splice site mutations, and large genomic deletions, which may be missed on traditional gene testing (Korf et al., 2015). Intronic splice site mutations are a main contributor to the lack of mutation identification in the TSC population with no identified mutations (Korf et al., 2015). Nowadays, NGS, whole exome sequencing (WES) or other multigene panels are wildly used in a larger number of TSC patients, including those with mosaicism and intronic variants affecting splicing (Peron et al., 2018). A comprehensive approach is now an effective method for detecting pathogenic variants in TSC. For example, Demir et al. (2021) utilized targeted high‐throughput sequencing and multiplex ligation‐dependent probe amplification (MLPA) methods to identify a mosaic likely pathogenic variant and the presence of a large genomic rearrangement in TSC. Similarly, Bąbol‐Pokora et al. (2021) employed a multistep molecular diagnostic strategy, including NGS, MLPA, and deep sequencing, to increase the likelihood of detecting potential variants for TSC and to enable the detection of mosaicism at low levels. Furthermore, minigene assay is an effective molecular diagnostic strategy for identifying intronic splice site variants. Our previous research using minigene assay identified a novel *TSC2* c.336_336 + 15delGGTAAGGCCCAGGGG variant in a TSC patient (Liu et al., 2022). Fan et al. identified a *TSC2* c.2742 + 5G>A variant using the same method (Fan et al., 2023). Similarly, our study conducted a minigene assay to confirm the pathogenicity of the variant.

In addition to the above‐mentioned studies, further investigations have addressed attention on the relationship between genotypes and phenotypes in TSC. Generally, *TSC2* variants are more common in patients with TSC and tend to cause more severe symptoms than *TSC1* variants (Au et al., 2007). Patients with *TSC2* variants usually present a higher number of tubers, an earlier age of seizure onset, and a higher prevalence of intellectual disability compared to those with *TSC1* variants (Curatolo et al., 2015). Compared with missense mutations, nonsense mutation or frameshift mutation might potentially manifest more severe TSC phenotypes in renal lesions (Muto et al., 2022). Additionally, the tendency to develop renal angiomyolipoma is higher in female patients than in male patients (Zhang et al., 2021). Moreover, different types and locations of TSC germline mutations may be associated with distinct neurocognitive phenotypes (van Eeghen et al., 2011). In this patient, the main clinical manifestations were hypomelanotic macules and ungual fibromas; hence, we speculate that the variant *TSC1*: c.1030‐2A>T is associated with a mild phenotype of TSC. Our result may be helpful in the diagnosis and genetic counseling of this TSC patient.

Because of similar clinical manifestations, TSC‐associated hypopigmentation may be initially confused with vitiligo. Earlier reports have described the existence of “vitiligo” and “depigmented nevi” in patients with TSC. Finally, it has been demonstrated that the hypopigmentation in TSC is different from that of vitiligo in that the number of melanocytes is not decreased in TSC hypopigmentation but is greatly decreased in vitiligo (Arbiser, 2015). The hypomelanotic macules that are characteristic of TSC mainly depend on the melanocytes of the TSC patients displaying autophagic dysregulation, thereby reducing the pigmentation (Yang et al., 2017). The use of 0.2% rapamycin gel showed substantial improvement in the hypopigmented macules, without systemic absorption (Wataya‐Kaneda et al., 2015). Hence, this targeted treatment process could represent a promising strategy for treating pigmentation disorders. Furthermore, rapamycin has been successful in treating animals with vitiligo (Chatterjee et al., 2014). Therefore, it might be a promising topical treatment for vitiligo in humans.

In conclusion, we identified a novel *TSC1* c.1030‐2A>T variant, which has not been previously documented in the Chinese or any other population. Additionally, the confirmation of its pathogenicity through a minigene splicing assay further unveiled the hidden TSC. This novel variant has not only broadened the mutant spectrum but also shed light on the complexity of TSC genetics and emphasized the importance of comprehensive genetic analysis in clinical practice. However, it is important to note that the study's focus on a single novel variant may limit the comprehensive understanding of the full spectrum of genetic variations associated with TSC. We look forward to more discoveries regarding the pathogenesis at the transcription and translation levels in the future, thus holding promise for the development of precision medicine strategies in TSC.

## AUTHOR CONTRIBUTIONS

*Clinical information collection*: Linli Liu and Yanbo Wang, *Sample collection and processing*: Linli Liu and Yanbo Wang. *Data analysis*: Linli Liu, Chunshui Yu, Zhengzhong Zhang. *Writing – original draft*: Linli Liu; Jin Chen. *Writing – review and editing*: Jin Chen. All authors have read and agreed to the published version of the manuscript.

## FUNDING INFORMATION

National Natural Science Foundation of China, Grant/Award Number: n82073462; Chongqing Science and Technology Commission, Grant/Award Number: 2023NSCQ‐MSX0321; Sichuan Medical Youth InnovationResearch Project, Grant/Award Number: Q23014.

## CONFLICT OF INTEREST STATEMENT

The authors declare no conflict of interest.

## ETHICS STATEMENT

This study was conducted according to the criteria of the Declaration of Helsinki and was approved and guided by The First Affiliated Hospital of Chongqing Medical University. Written informed consent was obtained from the patient for the publication.

## Data Availability

The data that support the findings of this study are available from the corresponding author upon reasonable request.

## References

[mgg32403-bib-0001] Ali Khan, I. (2021). Do second generation sequencing techniques identify documented genetic markers for neonatal diabetes mellitus? Heliyon, 7(9), e07903. 10.1016/j.heliyon.2021.e07903 34584998 PMC8455689

[mgg32403-bib-0002] Arbiser, J. L. (2015). Efficacy of rapamycin in tuberous sclerosis–associated hypopigmented macules. JAMA Dermatology, 151(7), 703–704. 10.1001/jamadermatol.2014.4299 25692853 PMC4496252

[mgg32403-bib-0003] Au, K. S. , Williams, A. T. , Roach, E. S. , Batchelor, L. , Sparagana, S. P. , Delgado, M. R. , Wheless, J. W. , Baumgartner, J. E. , Roa, B. B. , Wilson, C. M. , Smith‐Knuppel, T. K. , Cheung, M. Y. , Whittemore, V. H. , King, T. M. , & Northrup, H. (2007). Genotype/phenotype correlation in 325 individuals referred for a diagnosis of tuberous sclerosis complex in the United States. Genetics in Medicine, 9(2), 88–100. 10.1097/GIM.0b013e31803068c7 17304050

[mgg32403-bib-0004] Bąbol‐Pokora, K. , Bielska, M. , Bobeff, K. , Jatczak‐Pawlik, I. , Borkowska, J. , Kotulska, K. , Jóźwiak, S. , Młynarski, W. , & Trelińska, J. (2021). A multistep approach to the genotype‐phenotype analysis of polish patients with tuberous sclerosis complex. European Journal of Medical Genetics, 64(10), 104309. 10.1016/j.ejmg.2021.104309 34403804

[mgg32403-bib-0005] Chatterjee, S. , Eby, J. M. , Al‐Khami, A. A. , Soloshchenko, M. , Kang, H. K. , Kaur, N. , Naga, O. S. , Murali, A. , Nishimura, M. I. , Caroline Le Poole, I. , & Mehrotra, S. (2014). A quantitative increase in regulatory T cells controls development of vitiligo. The Journal of Investigative Dermatology, 134(5), 1285–1294. 10.1038/jid.2013.540 24366614 PMC3989443

[mgg32403-bib-0006] Crossley, B. M. , Bai, J. , Glaser, A. , Maes, R. , Porter, E. , Killian, M. L. , Clement, T. , & Toohey‐Kurth, K. (2020). Guidelines for sanger sequencing and molecular assay monitoring. Journal of Veterinary Diagnostic Investigation, 32(6), 767–775. 10.1177/1040638720905833 32070230 PMC7649556

[mgg32403-bib-0007] Curatolo, P. , Moavero, R. , Roberto, D. , & Graziola, F. (2015). Genotype/phenotype correlations in tuberous sclerosis complex. Seminars in Pediatric Neurology, 22(4), 259–273. 10.1016/j.spen.2015.10.002 26706013

[mgg32403-bib-0008] Demir, S. , Yalçıntepe, S. , Atlı, E. , Yalçın, Y. , Atlı, E. İ. , Eker, D. , Karal, Y. , & Gürkan, H. (2021). Comprehensive genetic analysis Results of TSC1/TSC2 genes in patients with clinical suspicion of tuberous sclerosis complex and definition of 3 novel variants. Balkan Medical Journal, 38(6), 341–347. 10.5152/balkanmedj.2021.21092 34860161 PMC8880961

[mgg32403-bib-0009] Ebrahimi‐Fakhari, D. , Meyer, S. , Vogt, T. , Pföhler, C. , & Müller, C. S. L. (2017). Dermatological manifestations of tuberous sclerosis complex (TSC). JDDG: Journal der Deutschen Dermatologischen Gesellschaft, 15(7), 695–700. 10.1111/ddg.13264 28598544

[mgg32403-bib-0010] Fan, K. , Guo, Y. , Song, Z. , Yuan, L. , Zheng, W. , Hu, X. , Gong, L. , & Deng, H. (2023). The TSC2 c.2742+5G>A variant causes variable splicing changes and clinical manifestations in a family with tuberous sclerosis complex. Frontiers in Molecular Neuroscience, 16, 1091323. 10.3389/fnmol.2023.1091323 37152430 PMC10157042

[mgg32403-bib-0011] Gaildrat, P. , Killian, A. , Martins, A. , Tournier, I. , Frébourg, T. , & Tosi, M. (2010). Use of splicing reporter minigene assay to evaluate the effect on splicing of unclassified genetic variants. Methods in Molecular Biology, 653, 249–257. 10.1007/978-1-60761-759-4_15 20721748

[mgg32403-bib-0012] Henske, E. P. , Jóźwiak, S. , Kingswood, J. C. , Sampson, J. R. , & Thiele, E. A. (2016). Tuberous sclerosis complex. Nature Reviews Disease Primers, 2, 16035. 10.1038/nrdp.2016.35 27226234

[mgg32403-bib-0013] Hung, C.‐C. , Su, Y.‐N. , Chien, S.‐C. , Liou, H.‐H. , Chen, C.‐C. , Chen, P.‐C. , Hsieh, C. J. , Chen, C. P. , Lee, W. T. , Lin, W. L. , & Lee, C.‐N. (2006). Molecular and clinical analyses of 84 patients with tuberous sclerosis complex. BMC Medical Genetics, 7, 72. 10.1186/1471-2350-7-72 16981987 PMC1592085

[mgg32403-bib-0014] Korf, B. R. , Tyburczy, M. E. , Dies, K. A. , Glass, J. , Camposano, S. , Chekaluk, Y. , Thorner, A. R. , Lin, L. , Krueger, D. , Franz, D. N. , Thiele, E. A. , Sahin, M. , & Kwiatkowski, D. J. (2015). Mosaic and intronic mutations in TSC1/TSC2 explain the majority of TSC patients with No mutation identified by conventional testing. PLoS Genetics, 11(11), e1005637. 10.1371/journal.pgen.1005637 26540169 PMC4634999

[mgg32403-bib-0015] Liu, L. , Yu, C. , & Yan, G. (2022). Identification of a novel heterozygous TSC2 splicing variant in a patient with tuberous sclerosis complex. Medicine, 101(3), e28666. 10.1097/md.0000000000028666 35060563 PMC8772658

[mgg32403-bib-0016] Lyczkowski, D. A. , Conant, K. D. , Pulsifer, M. B. , Jarrett, D. Y. , Grant, P. E. , Kwiatkowski, D. J. , & Thiele, E. A. (2007). Intrafamilial phenotypic variability in tuberous sclerosis complex. Child Neurology, 22, 1348–1355. 10.1177/0883073807307093 18174550

[mgg32403-bib-0017] Muto, Y. , Sasaki, H. , Sumitomo, M. , Inagaki, H. , Kato, M. , Kato, T. , Miyai, S. , Kurahashi, H. , & Shiroki, R. (2022). Genotype‐phenotype correlation of renal lesions in the tuberous sclerosis complex. Human Genome Variation, 9(1), 5. 10.1038/s41439-022-00181-1 35145067 PMC8831580

[mgg32403-bib-0018] Northrup, H. , Aronow, M. E. , Bebin, E. M. , Bissler, J. , Darling, T. N. , de Vries, P. J. , Frost, M. D. , Fuchs, Z. , Gosnell, E. S. , Gupta, N. , Jansen, A. C. , Jóźwiak, S. , Kingswood, J. C. , Knilans, T. K. , McCormack, F. X. , Pounders, A. , Roberds, S. L. , Rodriguez‐Buritica, D. F. , Roth, J. , … International Tuberous Sclerosis Complex Consensus Group . (2021). Updated international tuberous sclerosis complex diagnostic criteria and surveillance and management recommendations. Pediatric Neurology, 123, 50–66. 10.1016/j.pediatrneurol.2021.07.011 34399110

[mgg32403-bib-0019] Peron, A. , Au, K. S. , & Northrup, H. (2018). Genetics, genomics, and genotype–phenotype correlations of TSC: Insights for clinical practice. American Journal of Medical Genetics Part C: Seminars in Medical Genetics, 178(3), 281–290. 10.1002/ajmg.c.31651 30255984

[mgg32403-bib-0020] Randle, S. C. (2017). Tuberous sclerosis complex: A review. Pediatric Annals, 46(4), e166–e171. 10.3928/19382359-20170320-01 28414398

[mgg32403-bib-0021] Richards, S. , Aziz, N. , Bale, S. , Bick, D. , Das, S. , Gastier‐Foster, J. , Grody, W. W. , Hegde, M. , Lyon, E. , Spector, E. , Voelkerding, K. , Rehm, H. L. , & ACMG Laboratory Quality Assurance Committee . (2015). Standards and guidelines for the interpretation of sequence variants: A joint consensus recommendation of the American College of Medical Genetics and Genomics and the Association for Molecular Pathology. Genetics in Medicine, 17(5), 405–424. 10.1038/gim.2015.30 25741868 PMC4544753

[mgg32403-bib-0022] Roach, E. S. , & Sparagana, S. P. (2016). Diagnosis of tuberous sclerosis complex. Journal of Child Neurology, 19(9), 643–649. 10.1177/08830738040190090301 15563009

[mgg32403-bib-0023] Teng, J. M. C. , Cowen, E. W. , Wataya‐Kaneda, M. , Gosnell, E. S. , Witman, P. M. , Hebert, A. A. , Mlynarczyk, G. , Soltani, K. , & Darling, T. N. (2014). Dermatologic and dental aspects of the 2012 international tuberous sclerosis complex consensus statements. JAMA Dermatology, 150, 1095–1101. 10.1001/jamadermatol.2014.938 25029267 PMC11100257

[mgg32403-bib-0024] Togi, S. , Ura, H. , Hatanaka, H. , & Niida, Y. (2022). Genotype and phenotype landscape of 283 Japanese patients with tuberous sclerosis complex. International Journal of Molecular Sciences, 23(19), 11175. 10.3390/ijms231911175 36232477 PMC9569560

[mgg32403-bib-0025] Touraine, R. , Hauet, Q. , Harzallah, I. , & Baruteau, A. E. (2022). Tuberous sclerosis complex: Genetic counselling and perinatal follow‐up. Archives de Pédiatrie, 29(5), 5S3–5S7. 10.1016/s0929-693X(22)00283-4 36585068

[mgg32403-bib-0026] van Eeghen, A. M. , Black, M. E. , Pulsifer, M. B. , Kwiatkowski, D. J. , & Thiele, E. A. (2011). Genotype and cognitive phenotype of patients with tuberous sclerosis complex. European Journal of Human Genetics, 20(5), 510–515. 10.1038/ejhg.2011.241 22189265 PMC3330219

[mgg32403-bib-0027] Wataya‐Kaneda, M. , Tanaka, M. , Yang, L. , Yang, F. , Tsuruta, D. , Nakamura, A. , Matsumoto, S. , Hamasaki, T. , Tanemura, A. , & Katayama, I. (2015). Clinical and histologic analysis of the efficacy of topical rapamycin therapy against hypomelanotic macules in tuberous sclerosis complex. JAMA Dermatology, 151(7), 722–730. 10.1001/jamadermatol.2014.4298 25692384

[mgg32403-bib-0028] Yang, F. , Yang, L. , Wataya‐Kaneda, M. , Hasegawa, J. , Yoshimori, T. , Tanemura, A. , Tsuruta, D. , & Katayama, I. (2017). Dysregulation of autophagy in melanocytes contributes to hypopigmented macules in tuberous sclerosis complex. Journal of Dermatological Science, 89(2), 155–164. 10.1016/j.jdermsci.2017.11.002 29146131

[mgg32403-bib-0029] Zhang, N. , Wang, X. , Tang, Z. , Qiu, X. , Guo, Z. , Huang, D. , Xiong, H. , & Guo, Q. (2021). The correlation between tuberous sclerosis complex genotype and renal angiomyolipoma phenotype. Frontiers in Genetics, 11, 575750. 10.3389/fgene.2020.575750 33679864 PMC7933690

